# Effects of Different Corticosteroid Doses in Elderly Unvaccinated Patients with Severe to Critical COVID-19

**DOI:** 10.3390/life12111924

**Published:** 2022-11-18

**Authors:** Filippo Scialò, Domenica Francesca Mariniello, Ersilia Nigro, Klara Komici, Valentino Allocca, Andrea Bianco, Fabio Perrotta, Vito D’Agnano

**Affiliations:** 1 CEINGE, Biotecnologie Avanzate Scarl, 80145 Napoli, Italy; 2 Department of Translational Medical Sciences, University of Campania Luigi Vanvitelli, 81100 Naples, Italy; 3 Department of Environmental, Biological and Pharmaceutical Sciences and Technologies (DISTABIF), University of Campania Luigi Vanvitelli, Via Vivaldi 43, 81100 Caserta, Italy; 4 Department of Medicine and Health Sciences, University of Molise, 86100 Campobasso, Italy

**Keywords:** SARS-CoV-2, COVID-19, glucocorticosteroids

## Abstract

SARS-CoV-2 infection can induce a broad range of clinical symptoms, and the most severe cases are characterized by an uncontrolled inflammatory response with the overproduction of proinflammatory cytokines. Elevated levels of C-reactive protein, interleukin-1B, and interleukin-6 have become key signatures of severe COVID-19. For this reason, the use of 6 mg of dexamethasone has become a standard of care, although this regime may not be optimal. Even though various glucocorticoid doses have been proposed, it is still unclear which dose should be used to prevent adverse effects while at the same time reducing the inflammatory response. Here, we compared two different doses of corticosteroids in 52 elderly hospitalized patients with severe to critical COVID-19 to assess efficacy and safety. We showed that in patients receiving a higher dose of prednisone, the time to negative swab was significantly longer. Furthermore, although neither dose was correlated with the risk of death, patients receiving the high dose were more likely to have adverse events such as hyperglycemia, leukocytosis, an increase in systemic blood pressure, and others. Finally, the BMI, WBC number, and NLR value were directly related to death. In conclusion, although the optimal glucocorticoid dose is still undefined, our retrospective study supports the absence of beneficial effects in the utilization of higher doses of corticosteroids in elderly patients with severe to critical COVID-19.

## 1. Introduction

Severe acute respiratory syndrome coronavirus-2 (SARS-CoV-2), the cause of coronavirus disease 2019 (COVID-19), was first reported in Wuhan, China, in December 2019, and then moved rapidly across the globe with profound health and socioeconomic impacts [1]. Although SARS-CoV-2 infection can be asymptomatic or cause only mild symptoms in the majority of cases, it has been seen to progress to interstitial pneumonia and acute respiratory distress syndrome (ARDS) in nearly 10–20% of the cases that can, lastly, lead to death. In an infectious disease as heterogeneous as COVID-19, host factors are the key to determining disease severity and progression [2]. For severe COVID-19 disease, major risk factors include age, male sex, obesity, smoking, and underlying comorbidities such as hypertension, type 2 diabetes mellitus, pulmonary diseases, cancer, and others [3,4,5]. Evidence suggests that age itself is the most significant risk factor for severe COVID-19 disease and its adverse health outcomes [6]. As comorbidities often increase with age, aging itself has been strongly associated with worse outcomes, because of the pathophysiological changes that characterize the respiratory system [7,8]. Many patients with severe COVID-19 have an excessive inflammatory response caused by an uncontrolled release of proinflammatory cytokines, defined as a cytokine storm, causing diffuse alveolar damage [9]. The elevated levels of inflammatory markers, including C-reactive protein, ferritin, interleukin-1B, and interleukin-6 [10] represent the signature of a severe COVID-19 phenotype.

Glucocorticoid therapy has been a controversial issue in patients with SARS-CoV-2 infection; on the one hand, it can limit the inflammatory response, but, on the other hand, it has been feared that it may inhibit cell-mediated immunity, which can reduce viral clearance and worsen the course of the disease [11].

The RECOVERY trial shows that in patients hospitalized with COVID-19, the use of dexamethasone at a dose of 6 mg once daily, which is equivalent to 30 mg of methylprednisolone or 38 mg of prednisone [12], for up to 10 days compared to usual care resulted in a lower 28-day mortality among those who were receiving either invasive mechanical ventilation or oxygen alone, but not among those receiving no respiratory support [12]. Consequently, corticosteroid therapy has become the standard of care in critically ill patients with COVID-19, and was added to clinical practice guidelines [13,14]. The dose of 6 mg of dexamethasone is currently being reappraised and may miss important therapeutic potential, or may prevent the potential deleterious effects of higher doses of corticosteroids. Various glucocorticoid regimens have been proposed, and the optimal dose is still undefined and, in particular, few studies have focused on elderly patients, even though aging is a critical host factor to consider [15,16,17,18].

The aim of the study was to compare two different doses of corticosteroids in elderly hospitalized patients with severe to critical COVID-19 regarding efficacy and safety.

## 2. Materials and Methods

### 2.1. Patient Recruitment

A cohort of 52 patients with a diagnosis of severe to critical COVID-19 was recruited at U.O.C. Clinica Pneumologica “L. Vanvitelli” A.O. dei Colli—Ospedale Monaldi, Naples, Italy. Inclusion criteria were as follows: a positive rhinopharyngeal swab for SARS-CoV-2 RNA; age ≥65 years; evidence of clinical signs of COVID-19 pneumonia (fever, cough, dyspnea); plus one of the following: respiratory rate >30 breaths/min; severe respiratory distress; or SpO2 < 90% on room air [19]. Criteria for critical CODIV-19 were: (1) respiratory symptoms onset within 1 week; (2) chest imaging showing bilateral opacities not fully explained by volume overload; and (3) PaO_2_/FIO_2_ < 300 mmHg with PEEP or CPAP > 5 cmH_2_O [19]. Exclusion criteria were as follows: subjects vaccinated against SARS-COV-2; direct access to ICU for intensive support for respiratory failure; contraindications to corticosteroids. To limit the possible heterogeneity in the usage of different systemic glucocorticosteroids, we converted different agents to be prednisone equivalents according to the previous literature data [20].

The study was approved by the local ethics committee (n. 16223/2020, later amended) and was in accordance with the 1976 Declaration of Helsinki and its later amendments. Written consent was waived based on the observational study design, and to avoid SARS-CoV-2 contamination of the form and to limit the spread of the virus among the healthcare personnel; oral informed consent was obtained for the acquisition and treatment of clinical, laboratoristic, and imaging data.

### 2.2. Biochemical and Clinical Measurements

The anthropometric and biochemical features of total study participants are reported on in Table 1. The height and weight of patients were measured using standard techniques and the BMI was calculated as body weight (kg)/height^2^ (m^2^).

### 2.3. Statistical Analysis

Categorical data were expressed as numbers and percentages, while continuous variables as either a median and interquartile range or mean and standard deviation, according to the distribution assessed graphically and using the Shapiro–Wilk test. The presence of missing data was reported. The endpoint was in-hospital orotracheal intubation (IOT) or mortality, assessed either from data at discharge, IOT, or a death certificate. Univariable and multivariable logistic regression models were performed to evaluate the association between IOT and mortality with exposure variables. Odds ratios and 95% confidence intervals (OR—95% CI) were calculated for all models. A logistic regression analysis was performed to evaluate the presence of risk factors for the mentioned endpoint. The multivariable model was generated using forward selection and backward elimination processes, assessing all variables with a *p*-value < 0.25 in univariate analysis. The *p*-value for statistical significance was set at <0.05 for all the tests. All analyses were performed using statistical software STATA v16 (StataCorp. 2019. College Station, TX, USA: StataCorp LLC).

## 3. Results

### 3.1. Patient Characteristics

The anthropometric and biochemical characteristics of the 52 patients are reported on in Table 1. Patients were divided into two subgroups according to the prednisone equivalent use, < or ≥1 mg/kg; the first group included 14 participants, and the second one 38. The smoking status was similar in the two groups (35 vs. 24%), as well as the BMI (28 vs. 29). The comorbidities in the study population were, with a similar distribution between the two groups, as follows: systemic hypertension, CHD, atrial fibrillation, diabetes, and COPD. No statistical differences were present in the measured biochemical and inflammatory parameters (see Table 1).

### 3.2. Prednisone Equivalent Administration Determines the Time to Negative Swab

**Figure 1:**
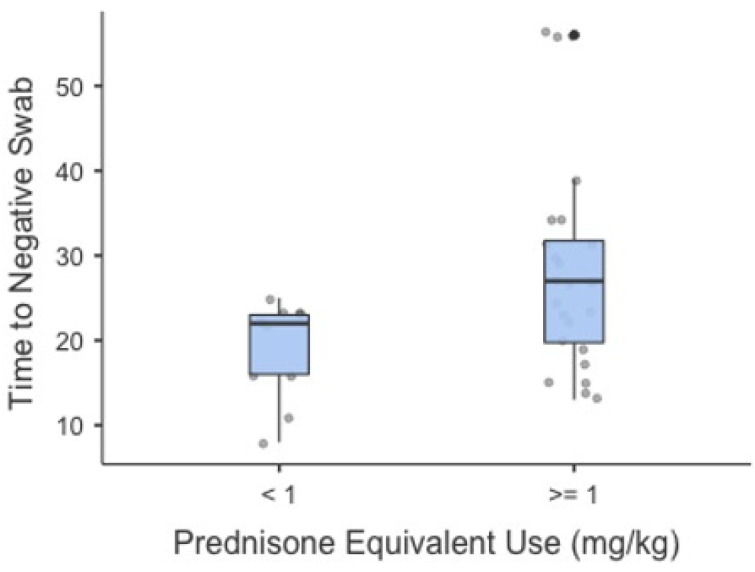
Time to negative swab in the two study populations. *p* = 0.0027. <1 and ≥1 refer to patients taking <1 mg/kg or ≥1 mg/kg prednisone.

Due to the possible influence on viral clearance, we considered the time to negative swab, finding that prednisone equivalent administration was directly correlated to it. Patients taking a dose of ≥1 mg/kg prednisone showed a longer time to negative swab (*p* > 0.027) compared to those taking <1 mg/kg (22 vs. 27 days) (Figure 1).

The time to clinical outcome is presented in Supplementary Table S1. We found a higher time to clinical outcome in patients treated with higher doses of corticosteroids among dismissed patients (*p* = 0.003). The corticosteroid dosages did not influence the time to clinical outcome among patients who experienced a negative course of the disease (*p* = 0.33).

### 3.3. The Risk of Death Is Not Related to Prednisone Equivalent Administration

We compared the risk of death among our patients according to several biochemical and clinical parameters and to prednisone equivalent administration (Table 2). The univariate analysis revealed that gender, age, smoking status, and prednisone equivalent administration did not influence the risk of death, while the BMI, WBC number, and NLR value were directly related to death (*p* < 0.05).

These data were confirmed in the final multivariate model (Table 3), showing that after being adjusted for potential confounding factors, only the BMI and Charlson comorbidity index were predictors of in-hospital death or IOT in the study population (*p* = 0.009 and 0.043, respectively).

## 4. Discussion

The results of this study suggested that a reduction in the dose of corticosteroids did not impact the risk of death in elderly hospitalized patients with severe to critical COVID-19. A possible explanation for the lack of significant differences in mortality derived from the use of different glucocorticoid doses could be due to the characteristics of these patients, as the two different groups both had advanced age and the same incidence of comorbidities, as demonstrated by the Charlson comorbidity index.

Older age is a predominant risk factor for severity and morbidity in patients with COVID-19 [6]. Immunosenescence represents a known feature of aging, with the disruption of both innate and adaptive arms of the immune system, and is considered to be the major reason for increased susceptibility to infection. In addition, the elderly exhibit a continual production of inflammatory mediators and cytokines, leading to the development of an exacerbated cytokine storm that may prompt an imbalance in the coagulative axis, resulting in fatal outcomes [21,22]. It may also influence ACE2 expression, the SARS-CoV-2 cell receptor, and facilitate viral entry [23]. Finally, the evidence suggests an aberrant ciliary function and ciliary ultrastructural anomalies with an age-related decline in the clearance of SARS-CoV-2 particles [24].

It is worth considering that our study population was composed of unvaccinated patients, showing a severe phenotype not attenuated by vaccines. Indeed, the vaccines, especially mRNA ones, elicit polyfunctional antibodies that mediate virus neutralization and a potent T cell response (Immunological mechanisms of vaccine-induced protection, 2021, M Sadarangani). These events dramatically reduce the severe manifestations of COVID-19; thus, patients rarely require invasive care. On the contrary, in unvaccinated patients, in consideration of the high rate of severe events, the optimization of therapeutic options still attracts considerable medical interest.

Without evidence of the superior efficacy of high-dose corticosteroids, it seems prudent to use the minimal effective dose until more data are available [25]. In elderly patients, the benefit of corticosteroid therapy may be outweighed by metabolic side effects, which can precipitate pre-existing comorbidities, including hypertension, diabetes, risk of bone fractures, psychiatric alterations, and cataracts [19]. In fact, the risk of experiencing adverse effects, such as hyperglycemia, leukocytosis, an increase in systemic blood pressure, arrhythmia, and others, in the study population was not influenced by different GC regimens (Supplementary Table S1). Although dexamethasone has demonstrated a promising performance for severe COVID-19, there are still concerns regarding its prescription for indiscriminate cases, because dexamethasone can disturb the development of the host’s natural immunity and abrogate the antiviral response, which could lead to a delayed viral clearance.

Indeed, delayed SARS-CoV-2 clearance in corticosteroid-treated patients was found in previous reports in patients with SARS-CoV-2 [26] and MERS-CoV-2 [27] infections. The altered viral clearance may be related to corticosteroid effects on T cell responses and on interferon pathways [28]. Our data confirmed these data, as demonstrated by the time to negative swab, which was statistically delayed in patients taking higher doses of glucocorticoids.

Safety is the main concern of corticosteroid therapy in patients with COVID-19. Besides the previously discussed impairment of viral clearance [29], the increased risk of secondary infections associated with high doses of systemic GCs was noticed [30,31]. As discussed worldwide, diabetes is a major risk factor for severe COVID-19, and treatment with dexamethasone could be associated with the development of hyperglycemic conditions [32,33,34]. Hyperglycemia is a dose-dependent adverse effect of corticosteroid therapy, favoring the infection and the severity of the disease [35]. Furthermore, hypertensive patients should be under constant monitoring during dexamethasone treatment once GC-induced hypertension is observed [36]. High dexamethasone doses are associated with increased sodium retention, which leads to the elevation of blood pressure, in addition to the chemical alteration of peripheral nerve homeostasis [30,37,38].

In our population study, a predictor of in-hospital mortality was the BMI. Obesity was an independent risk factor for death in all age groups [39,40]. It is known that the level of the expression of ACE2 in adipose tissue is higher than in lung tissue, so patients with obesity express a high number of ACE2 receptors [41]. In addition, obesity impairs cytokine expression, resulting in an impaired immune response [42].

Men are well known to be infected more frequently than women, and are more likely to develop more severe disease [3,4,10]. Sex disparities in COVID-19 severity and mortality are multifactorial. Globally, men have more comorbidities than women [43]. Sex differences could be involved in viral entry. Interestingly, a study by Asselta et al. (2020), which compared the expression of transmembrane protease serine 2 (TMPRSS2), crucial for viral entry [44], in the two sexes of a large Italian cohort, observed a higher expression of TMPRSS2 in bronchial epithelial cells in the males compared to females [45]. Additionally, sex-based differences in immune responses have been reported on [46]. Females differ in their innate recognition and response to viral infections and mount a greater cellular and humoral immune response [47]. Additionally, sex hormones regulate immunity, and are likely to play a role in differences in the severity of COVID-19 between males and females. Ding et al. (2020) showed that postmenopausal women were at a greater risk of hospitalization, and that estrogen levels had a protective effect against disease severity [48]. This protective effect of estrogen was attributed to reduced levels of inflammatory cytokines, such as IL-6, IL-8, and TNFα42.

Regarding the laboratory tests, in our population study, the leukocyte count and neutrophil–lymphocyte ratio (NLR) were statistically higher in nonsurvivors than in survivors. Our results showed a similar tendency compared with other studies. Most severe COVID-19 cases presented low lymphocyte counts and high leukocyte counts and NLR, as well as lower percentages of monocytes, eosinophils, and basophils [49]. Several studies have suggested the presence of a prognostic role of the NLR in various inflammatory diseases and oncological processes [50,51,52]. Normal NLR values in an adult in good health have been reported to be between 0.78 and 3.53, and it is a simple parameter to easily assess the inflammatory status of a subject [53]. The NLR appears to be an independent marker of systemic endothelial dysfunction [54,55]. The NLR may be useful in identifying hospitalized COVID-19 patients with poor prognoses.

It is well known that corticosteroids have several effects on the response of the immune system, classically producing lymphopenia and neutrophilia, and also decreasing cytokine production. In theory, this mechanism may explain the increase in the NLR. However, various studies regarding the prognostic value of the NLR in inflammatory diseases have shown a reduction in the ratio in patients under corticosteroid treatment [56].

Many studies have reported on other prognostic markers for SARS-CoV-2, such as leukocytosis and C-reactive protein (CRP) [57], and blood urea nitrogen combined with D-dimer [4]. In our study, the D-dimer level was not different between survivors and the dead. All patients used low-molecular-weight heparin (LMWH) at a prophylactic or therapeutic dose. Respiratory failure was the most common cause of death, but coagulation activation accompanied by excessive immune/inflammatory reactions, thrombosis and disseminated intravascular coagulation (DIC), and progression to multiorgan failure were also causes of death. The most frequently described report related to COVID-19 coagulopathy was an increase in plasma D-dimer levels [58,59,60].

Our study had some limitations. Firstly, a few patients were treated with a corticosteroid different from methylprednisolone, which may have had different pharmacokinetic properties when compared to dexamethasone; however, the intravenous administration and the use of a prednisone equivalent dose limited this bias. Additionally, because of the pragmatic nature of this retrospective study design, including elderly hospitalized patients with severe to critical COVID-19, we could not exclude whether patients before hospitalization may have been treated differently, and that this could have had an impact on the final clinical outcomes. Finally, the time to negative swab was assumed based only on the negative SARS-CoV-2 RT-PCR test results, regardless of the cycle threshold value that could have offered more robust evidence on viral clearance. Therefore, further studies in larger cohorts are necessary to confirm our preliminary data.

## 5. Conclusions

In conclusion, the optimal dose is still not clear, but low-dose corticosteroid therapy is recommended as the standard of care for hospitalized patients with COVID-19 who require supplemental oxygen, in particular for the elderly. Higher doses of corticosteroids may offer additional anti-inflammatory effects, but may also be associated with a higher risk of serious adverse events in severe and critically ill elderly COVID-19 patients.

## Figures and Tables

**Table 1:** Study population baseline characteristics.

	Prednisone Equivalent Use		
	<1 (mg/kg) (n = 14)	≥1 (mg/kg) (n = 38)	*p*
Age	68 (68–81.8)	74.5 (70.3–78.8)	0.185
Gender (Male)	9 (64.2)	18 (47.4)	0.278
BMI	27.7 (25.7–29)	29.2 (26–35)	0.235
Smoking	5 (35.7)	9 (23.7)	0.485
Comorbidities			
Systemic Hypertension	8 (57.1)	24 (63.2)	-
CHD	2 (14.3)	12 (31.6)	-
Atrial Fibrillation	2 (14.3)	5 (13.2)	-
Diabetes	4 (28.6)	12 (31.6)	-
COPD	2 (14.3)	9 (23.7)	-
Charlson Comorbidity Index	3 (2.25–4)	4 (3–4)	-
Time to Negative Swab (d)	22 (16–23)	27 (19.8–31.8)	0.027
LUS SCORE	32 (27.3–33)	30 (24–36	0.77
CHUNG SCORE	15 (11.5–15)	14 (12.3–15.8)	0.942
Corticosteroids			
Methylprednisolone	12 (85.7)	38 (100)	-
Dexamethasone	2 (14.3)	-	-
Anticoagulants			
Prophylactic LMWH	9 (64.3)	24 (63.2)	-
Therapeutic LMWH	3 (21.4)	14 (36.8)	-
NAO	2 (14.3)	-	-
Remdesivir	6 (42.8)	13 (34.2)	-
Respiratory Support			
Venturi or Non-Rebreathing Mask	2 (14.3)	1 (2.6)	-
HFNC	2 (14.3)	8 (21.0)	-
CPAP	6 (42.9)	23 (60.5)	-
NIV	4 (28.6)	6 (15.8)	-
WBC	7.05 (4.09-11.1)	8.79 (6.9–12.6)	0.111
Neutrophils	6.13 (3.12–10.1)	7.23 (6.31–11.4)	0.104
Lymphocytes	0.51 (0.44–0.82)	0.73 (0.45–1.14)	0.375
RBC	4.5 (3.77–4.62)	4.64 (4.33–5.1)	0.434
HGB	12.3 (11.1–13.8)	12.8 (9.6–14.3)	0.735
PLT	247 (199–275)	211 (159–243)	0.078
D-Dimer	525 (419–1440)	456 (278–613)	0.383
CRP	6.2 (3.22–7.3)	6.8 (2.25–10.9)	0.542
Creatinine	0.8 (0.6–0.87)	0.7 (0.6–0.9)	1
Na+	139 (136–144)	138 (136–140)	0.285
K+	4.25 (3.7–4.7)	4.1 (3.77–4.63)	0.853
AST	61 (26.3-69.8)	25 (18.3–43.8)	0.024
ALT	63 (20.5–89.5)	27 (17.3–32)	0.06
LDH	386 (277–568)	387 (292–509)	0.754
Albumin	4.1 (3.3–4.1)	3.7 (3.2–4.2)	0.693
IL-6	47.8 (20.6–151)	71.4 (15.6–126)	0.864
KL-6	668 (502–1017)	931 (482–1750)	0.57
NT-PRO-BNP	139 (119–932)	346 (85.3–454)	0.527
P/F	120 (85–138)	91 (84–120)	0.135
Lac	1 (0.95–1.2)	1.2 (1–1.33)	0.094
Death	5 (35.7)	14 (36.8)	0.94

Data are presented as median (IQR) or absolute number (%). CPAP: continuous positive airway pressure; d: days; HFNC: high-flow nasal cannula; LMWH: low-molecular-weight heparin; NAO: oral anticoagulant; NIV: noninvasive ventilation.

**Table 2:** Univariate analysis for risk of death among elderly with severe to critical COVID-19.

	Survivors (n = 33)	Deaths (n = 19)	*p*
Gender (Male)	14 (42.4)	6 (31.6)	0.558
Age	73 (69–78)	78 (67.5–84.5)	0.233
BMI	27.7 (25.1–29.4)	32.5 (28.5–35.2)	0.001
Charlson Comorbidity Index	3 (3–4)	4 (4–4)	0.113
Smoking (Yes)	9 (27.3)	5 (26.3)	1.000
CHUNG SCORE	14(12–15)	15 (12.5–16)	0.060
Prednisone Equivalent (<1 mg/kg)	9 (27.3)	5 (26.3)	1.000
WBC	7.3 (5.9–9.5)	11.3 (8.7–12.6)	0.020
NLR	9.1 (5.3–14.5)	15.5 (10.4–15.6)	0.017
D-Dimer	426 (210–619)	512 (456–936)	0.061
P/F	91 (84–127)	100 (93–124)	0.624

**Table 3:** Multivariate analysis for risk of death among elderly with severe to critical COVID-19.

		95% Confidence Interval			
Predictor	Estimate	Lower	Upper	SE	*p*
Prednisone Equivalent Use	−1.947	−44.272	0.532	12.652	0.124
BMI	0.363	0.0892	0.636	0.1395	0.009
CHUNG-SCORE	0.197	−0.2270	0.621	0.2164	0.362
NLR	0.137	−0.0391	0.312	0.0897	0.128
D-Dimer	1.14 × 10^−5^	−1.98 × 10^−4^	2.21 × 10^−4^	1.07 × 10^−4^	0.915
Charlson Comorbidity Index	1.198	0.0370	2.359	0.5922	0.043

## Data Availability

Data are available upon request.

## Supplementary Materials

The following supporting information can be downloaded at: https://www.mdpi.com/article/10.3390/life12111924/s1, Table S1: Difference in days from hospital admission to clinical outcome according prednisone equivalent dose.
