# Supplementary material for: Effects of Different Corticosteroid Doses in Elderly Unvaccinated Patients with Severe to Critical COVID-19
Source: Life (Basel). 2022 Nov 18;12(11):1924. doi: 10.3390/life12111924 (PMC9697502; doi:10.3390/life12111924)
Supplement: Supplementary file 1 [file life-12-01924-s001.zip › life-1980930-supplementary.pdf]

Supplementary Table S1: Difference in days from hospital admission to clinical outcome according prednisone equivalent dose.

	Dismissed patients			Deceased or IOT patients		
**Prednisone Equivalent Use (mg/kg)**	Mean	SD	p	Mean	SD	p
< 1	19.78	5.43	0.038	20.00	10.25	0.33
≥ 1	29.17	12.49		26.57	13.09	

Data are presented as mean value± standard deviation (SD).
